# Energy expenses on prey processing are comparable, but paid at a higher metabolic scope and for a longer time in ambush vs active predators: a multispecies study on snakes

**DOI:** 10.1007/s00442-021-05014-6

**Published:** 2021-08-14

**Authors:** Stanisław Bury

**Affiliations:** Department of Comparative Anatomy, Institute of Zoology and Biomedical Research, Jagiellonian University, Gronostajowa 9, 30-387 Kraków, Poland

**Keywords:** Snakes, Metabolism, Digestion, Foraging, SDA, Sit-and-wait predator

## Abstract

Snakes are characterized by distinct foraging strategies, from ambush to active hunting, which can be predicted to substantially affect the energy budget as a result of differential activity rates and feeding frequencies. Intense foraging activity and continuously upregulated viscera as a result of frequent feeding leads to a higher standard metabolic rate (SMR) in active than in ambush predators. Conversely, the costs of digestion (Specific Dynamic Action—SDA) are expected to be higher in ambush predators following the substantial remodelling of the gut upon ingestion of a meal after a long fasting period. This prediction was tested on an interspecific scale using a large multispecies dataset (> 40 species) obtained from published sources. I found that the metabolic scope and duration of SDA tended to reach higher values in ambush than in active predators, which probably reflects the greater magnitude of postprandial physiological upregulation in the former. In contrast, the SDA energy expenditure appeared to be unrelated to the foraging mode. The costs of visceral activation conceivably are not negligible, but represent a minor part of the total costs of digestion, possibly not large enough to elicit a foraging-mode driven variation in SDA energy expenditure. Non-mutually exclusive is that the higher costs of structural upregulation in ambush predators are balanced by the improved, thus potentially less expensive, functional performance of the more efficient intestines. I finally suggest that ambush predators may be less susceptible than active predators to the metabolic ‘meltdown effect’ driven by climate change.

**Supplementary Information:**

The online version contains supplementary material available at 10.1007/s00442-021-05014-6.

## Introduction

Limitations in the availability of food resources represent a powerful selective force, and its importance in the evolution of organisms was pointed out already by Darwin in his magnum opus “*On the origin of species*” (Darwin 1859). Food resources are necessary for growth (Bury 2021; Dunham 1978), maintenance (Møller et al. 1998; Speakman and McQueenie 1996) and reproduction (Warner et al. 2008; Lindström et al. 2005), so maximized resource intake might seem most profitable for fitness. However, a high resource intake requires greater foraging activity (Secor and Nagy 1994; Werner and Anholt 1993), which is associated with extended exposure to predators (Webb et al. 2003; Werner and Anholt 1993) and elevated costs of self-maintenance owing to prolonged periods of high metabolic activity in tissues (Stuginski et al. 2018a; Secor and Diamond 2000). This indicates that the acquisition of food resources is subject to an evolutionary trade-off whereby the benefits of frequent feeding associated with large amounts of food consumed can be offset by higher predation and energy expenditure and vice-versa: the benefits of less risky and less energy-consuming, infrequent feeding are balanced by the cost of smaller amounts of ingested food (Abrams 1991, 1982). Under such a trade-off, a continuum of foraging strategies can be expected to have evolved, with frequently feeding active predators on the one hand, and infrequently feeding, sedentary ambush predators on the other. Among terrestrial vertebrates, snakes are particularly well documented as being characterized by such distinct modes of foraging (Lourdais et al. 2014; Beaupre and Montgomery 2007). These foraging modes in snakes have evolved convergently in different phylogenetic lineages and in a wide array of habitats, making snakes a useful and robust model for studying the ecological and physiological consequences of foraging modes (Glaudas et al. 2019).

The feeding rate specific to a foraging mode can be translated into the different amounts of energy gained within a period of time and the various duration of fasting between meals (Secor and Diamond 2000). Consequently, these differences must be associated with a corresponding variation in energy budgets (syndrome hypothesis—Beaupre and Montgomery 2007). Relatively short fasting periods due to frequent feeding and greater movement intensity in active than in ambush predators are proposed to result in continuously maintained high tissue activity and physiological readiness (Secor and Diamond 2000). This should elevate the costs of self-maintenance—expressed as the standard metabolic rate (SMR)—in active rather than ambush predators, a pattern indeed initially shown in a limited number of species (Secor and Diamond 2000), but recently confirmed by large multispecies datasets within a phylogenetic framework (Stuginski et al. 2018a; Dupoué et al. 2017). Physiological quiescence as expressed by a lower SMR in ambush predators during extended fasting thus appears to be a beneficial energy-saving strategy. When a meal is ingested, however, the activation of the downregulated digestive tract in ambush predators is hypothesized to entail a side-effect of energetic costs (‘pay-before-pumping’ effect; Secor and Diamond 1995) that may represent a substantial component of the total expenditure for meal processing (Specific Dynamic Action—SDA; Secor 2001, 2009). As a result, the various measures describing the costs of digestion, i.e. the amount of energy above SMR expended during digestion, SDA duration, and the range of the metabolic rate increase, may be higher in ambush than in active predators (Beaupre and Montgomery 2007; Secor and Diamond 2000). Besides the ‘pay-before-pumping’ model higher values of SDA variables in ambush predators to are sometimes discussed as an adaptation to feeding on larger average meal size (e.g. Secor et al. 1994). Larger meals elevate the costs of digestion which is a pattern widely shown in snake species representing both foraging modes (Bessler et al. 2010; Toledo et al. 2003; Zaidan and Beaupre 2003). The ‘meal size’ hypothesis suggests, however, that the costs of food processing paid by ambush predators exceed the expenses that could be predicted based on the sole effect of a meal size. An excessive amounts of energy spent on processing larger prey would represent a wasteful solution, definitely not beneficial given long periods of food deprivation in infrequently feeding ambush predators. Natural selection tends to favour frugal strategies (Even and Nicolaïdis 1993; Harshman et al. 1999; Dulloo and Girardier 1990; Szarski 1983), thus would rather lead to attenuation of the costs that are already high and regularly experienced due large sizes of most ingested meals. Consequently, if SDA in ambush predators is adapted to a larger meal size then rather a smaller costs of digestion should be expressed by ambush than active predators after ingesting a meal of a comparable size. However, given that recent study on a wide range of species have shown that foraging mode is not related to the average meal size (Glaudas et al. 2019), this ‘meal size’ hypothesis is no longer grounded in the ecological data.

So far, the comparative approach to test the effect of foraging mode on SDA in has been applied probably only by Secor and Diamond (2000), and solely on a small number of species of limited phylogenetic diversity, which precludes drawing general conclusions regarding the foraging-mode-specific costs of digestion. Those authors pointed out that an improved phylogeny and additional species would need to be studied (Secor and Diamond 2000). The potential results could shed more light on the role of foraging mode in species decline (see Reading et al. 2010), especially as the availability of food resources is predicted to shrink whereas energy expenditures are expected to rise as a result of global climate change (Huey and Kingsolver 2019). In this study I tested whether the costs of digestion in snakes, i.e. SDA, were syndromic to foraging mode (Beaupre and Montgomery 2007). In accordance with the ‘pay-before-pumping’ hypothesis (Secor and Diamond 1995), I predicted that the characteristics of SDA were correlated with the hunting strategy, i.e. that they would reach higher values in ambush than in active predators. As measures of the costs of digestion, I used three variables that describe SDA—energy expenditure, metabolic scope and duration. SDA energy expenditure and metabolic scope refer to costs at the level of energy metabolism, while SDA duration defines the costs in terms of the time needed to complete SDA response. SDA has been measured in many species of snakes representing various phylogenetic lineages, but most reports have focused only on a single species; a higher number of species has only rarely been investigated simultaneously (e.g. Stuginski et al. 2018b; Secor and Diamond 2000; Bedford 1996). Hence, this is the first study to apply a phylogenetically informed comparative framework to a large multispecies dataset that collates all the available data.

## Materials and methods

**Fig. 1:**
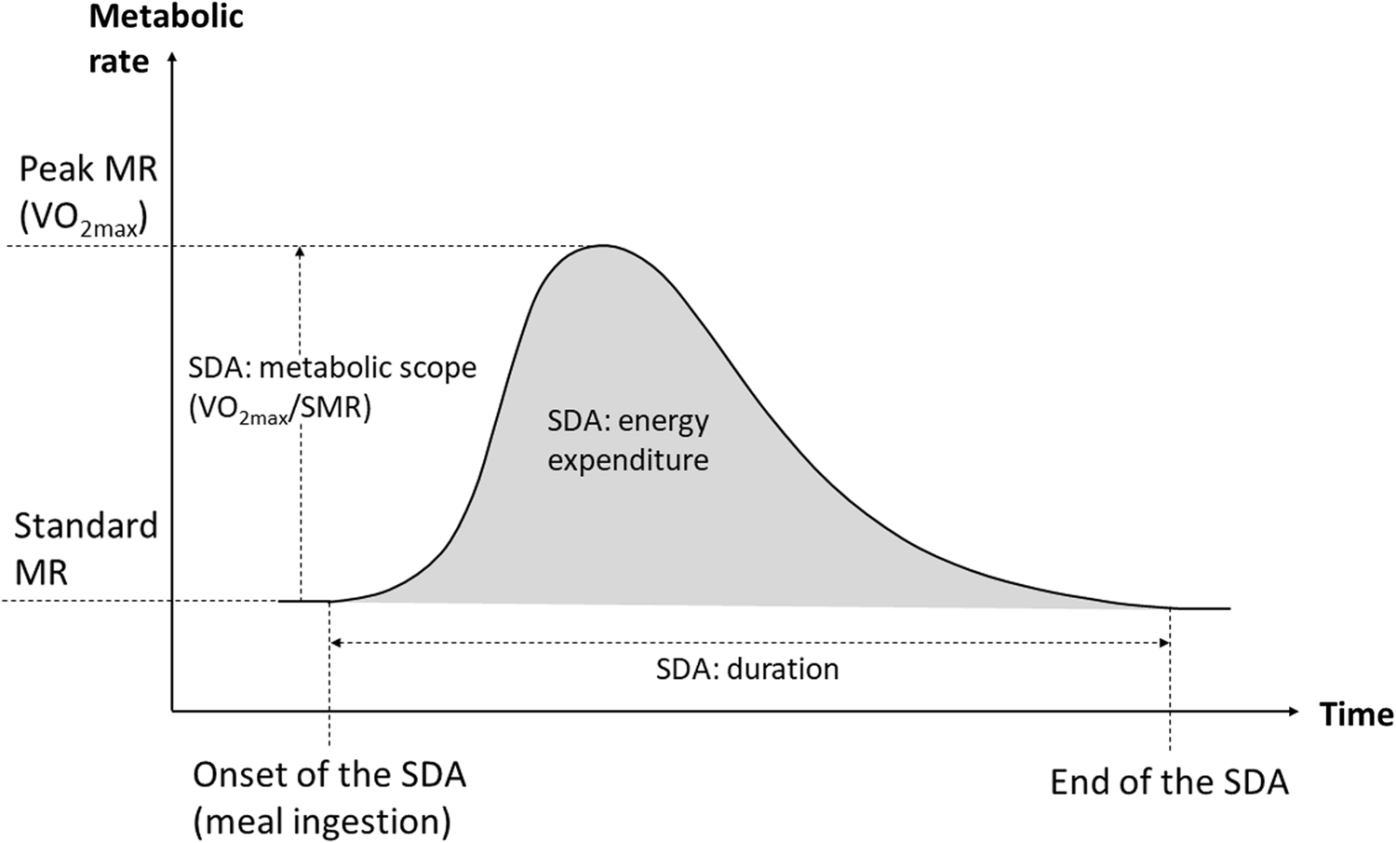
Typical response of metabolic rate (MR) to ingestion of a meal, i.e. Specific Dynamic Action (SDA). Variables studied here are as follows: SDA: energy expenditure—total amount of energy above Standard MR expended on meal processing. SDA: metabolic scope—factorial increase of MR at the peak of SDA (the ratio VO_xmax_/Standard MR). SDA: duration—time between the onset of the SDA (meal ingestion; when MR rises above SMR) and the end of the SDA (when MR returns to the level of SMR)

The dataset used in this study was compiled from published sources similarly as in other studies on snake metabolic rates (e.g. Stuginski et al. 2018a, b; Dupoué et al. 2017). The core dataset was obtained from the summary in Secor (2009) and supplemented with data from more than ten original studies not included in Secor (2009): the final dataset and reference list are given in the Supplementary Materials. I extracted data on three SDA-related variables that provided a good description of the costs of digestion and had the highest representation in terms of the number of species for which they were assessed. These variables were: SDA energy expenditure (SDA_expenditure_)—the amount of energy expended on digestion above the level of SMR, expressed in kJ (*N* = 44 species); SDA metabolic scope (SDA_scope_)—a factorial increase in the metabolic rate, expressed as the ratio of the peak metabolic rate during SDA to SMR (*N* = 46 species); SDA duration (SDA_duration_)—the period of time for which the metabolic rate remained above the level of SMR, expressed in days (*N* = 43 species) (see Fig. 1; Secor 2009). In some studies, SDA_expenditure_ was expressed in ml of O_2_ (e.g. Tsai et al. 2008), and these values were converted into kJ, assuming 19.8 J expended per ml of O_2_ according to other studies on snakes (e.g. Stuginski et al. 2018b; Secor and Diamond 2000). I included only those records where snakes were fed with fish or mammalian prey. This is because these two types of prey elicit a highly comparable SDA response, whereas amphibian and non-vertebrate meals generate a clearly smaller response (Bessler et al. 2010). Therefore, snakes of the genus *Dasypeltis* were removed entirely from the dataset because they were fed exclusively with eggs (Greene et al. 2013; Großmann and Starck 2006). As SDA is known to be affected by the size of a snake, the relative size of a meal provided and the measurement temperature (e.g. Zaidan and Beaupre 2003), all these variables were extracted along with SDA parameters. Snakes were assigned as being ambush (sit-and-wait; infrequently feeding) or active (frequently feeding) predators on the basis of the original studies from which I extracted the data. If no information on the foraging mode was given, I performed an additional literature search; the references in which the foraging mode was assessed are also provided in the Supplementary Materials. Since more than one record was available for many species, I averaged the values of the response variables and predictors for the purpose of this analysis. The risk of a potential bias in the obtained results needs to be mentioned, owing that different authors can use various approaches in measuring and estimating metabolic rates, particularly SMR. First, there are different techniques used to measure metabolic rates, with open-flow and closed-system respirometry being the most common. The respirometry method was, however, shown not to drive the variation in SMR (Dupoué et al. 2017). Second, the value of SMR can be estimated in a multiple ways, most widely as the single lowest MR value recorded (e.g. Secor and Diamond 2000) or the average of several lowest MR values recorded over a given period of time (Chu et al. 2009). The majority of studies on snake SDA uses approach of Secor and Diamond (2000), i.e. express SMR as the one lowest MR records, not the average. Metabolic rate is a significantly repeatable trait (Nespolo and Franco 2007), therefore, using averaged value instead of a single value should produce a comparable results. Finally, it is not possible to standardize for the effect of MR estimation method, because some studies do not provide enough details. These studies, however, cover a wide representation of species of both foraging modes (e.g. Bedford 1996), thus their methodological approach in MR assessment does not interfere with the main effects tested here.

To account for the non-independence among the species in the dataset emerging from their phylogenetic relationships, I employed a phylogenetic informed approach to the analysis. I used the comprehensive phylogeny of squamates that includes branch lengths (Pyron et al. 2013). Because the phylogeny contains more than 4000 squamate species, I pruned the tree to include the species in the dataset using *drop.tip* function in the package ‘ape’ (Paradis et al. 2021). One species was not included in the phylogeny (*Pseudonaja nuchalis*), so I used the position of its nearest sister taxon in the phylogeny, i.e. *Pseudonaja textilis* (see also Stuginski et al. (2018a). I tested for differences in the three attributes of SDA among foraging modes using the function *phylolm* in the package ‘phylolm’ (Ho et al. 2018) under a lambda model of phylogenetic covariance (Freckleton et al. 2002; Grafen 1989). I included snake body mass, relative meal size and measurement temperature as covariates, next to the foraging mode. Data on snake body mass and SDA_expenditure_ were log-transformed prior to the analyses. Given that those additional predictors (snake body mass, relative meal size and measurement temperature) appeared to have significant effect on SDA variables in most cases I performed additional univariate analysis. Specifically, I first extracted residual values of each SDA variable from the models that included side-factors, i.e. body mass, meal size and measurement temperature (function *residuals.phylolm*). The obtained residual values independent to the effects of these covariates were then used included in a PGLS model with foraging mode as the only factor.

## Results

Multivariate PGLS regression models showed that the effect of foraging mode was significant for two of the three variables tested—Table 1 sets out a detailed report of these models. No effect of the foraging mode on SDA_expenditure_ was detected, indicating that both, active and ambush predators, expend similar amounts of energy on meal processing (*p* = 0.89; Table 1). In contrast, the effect of foraging mode was detected for SDA_scope_ (*p* = 0.04; Table 1), which tended to take lower values in active than in ambush predators. Similar and significant association to foraging mode was detected for SDA_duration_ in that SDA lasts for a shorter time in active compared to ambush hunters (*p* < 0.01; Table 1). The effects of additional factors included in the model also differed between the response variables. The two variables related to the metabolic costs of digestion, i.e. SDA_expenditure_ and SDA_scope_, were positively correlated with snake body mass (*p* < 0.01 and *p* = 0.04, respectively; Table 1) and relative meal size (*p* < 0.05 and *p* = 0.02, respectively; Table 1), but no significant effect was found for temperature (*p* = 0.82 and *p* = 0.43, respectively; Table 1). SDA_duration_ was in turn unaffected by a snake’s size (*p* = 0.21; Table 1), but depended positively on meal size (*p* < 0.01) and negatively on temperature (*p* < 0.01; Table 1).

**Table 1:** Results of three PGLS models testing for the effects of foraging mode on three variables describing Specific Dynamic Action (SDA) in snakes

Response variable	No. of species	Lambda (λ)	*R*^2^	Effect	Estimate	Standard error	*t* value	*p* value
SDA: energy expenditure [log(kJ)]	44	0.126	0.853	Foraging mode [ambush vs active]	− 0.014	0.102	− 0.141	0.889
				Body mass [log(g)]	1.117	0.092	12.141	< 0.01***
				Meal size [% of body mass]	0.016	0.008	2.071	0.045*
				Measurement temperature [℃]	0.005	0.021	0.235	0.816
SDA: peak metabolic scope [VO_2_max/SMR]	46	1	0.291	Foraging mode [ambush vs active]	− 2.170	1.028	− 2.112	0.041*
				Body mass [log(g)]	1.205	0.574	2.099	0.042*
				Meal size [% of body mass]	0.105	0.044	2.394	0.021*
				Measurement temperature [℃]	0.103	0.129	0.793	0.432
SDA: duration [no. of days]	43	< 0.01	0.658	Foraging mode [ambush vs active]	− 2.257	0.567	− 3.978	0.0003***
				Body mass [log(g)]	0.674	0.529	1.275	0.210
				Meal size [% of body mass]	0.158	0.048	3.269	0.002**
				Measurement temperature [℃]	− 0.767	0.129	− 5.941	< 0.01***

The effects of body mass, meal size and temperature have been included in each model. *p *values indicating significant effects are depicted by asterisks (***p < 0.001; **p < 0.01; *p < 0.05)

Univariate approach with residual values of SDA variables independent to relative meal size, snake body mass and measurement temperature showed a similar results. Specifically, residual of SDA_expenditure_ was unrelated to the foraging mode (*t* =  − 0.15; *p* = 0.88; Fig. 3a), while residual values of SDA_scope_ (*t* =  − 2.18, *p* = 0.03; Fig. 3b) and SDA_duration_ (*t* =  − 4.07; *p* < 0.01; Fig. 3c) were higher in ambush compared to active predators.

## Discussion

### Foraging mode and SDA

**Fig. 2:**
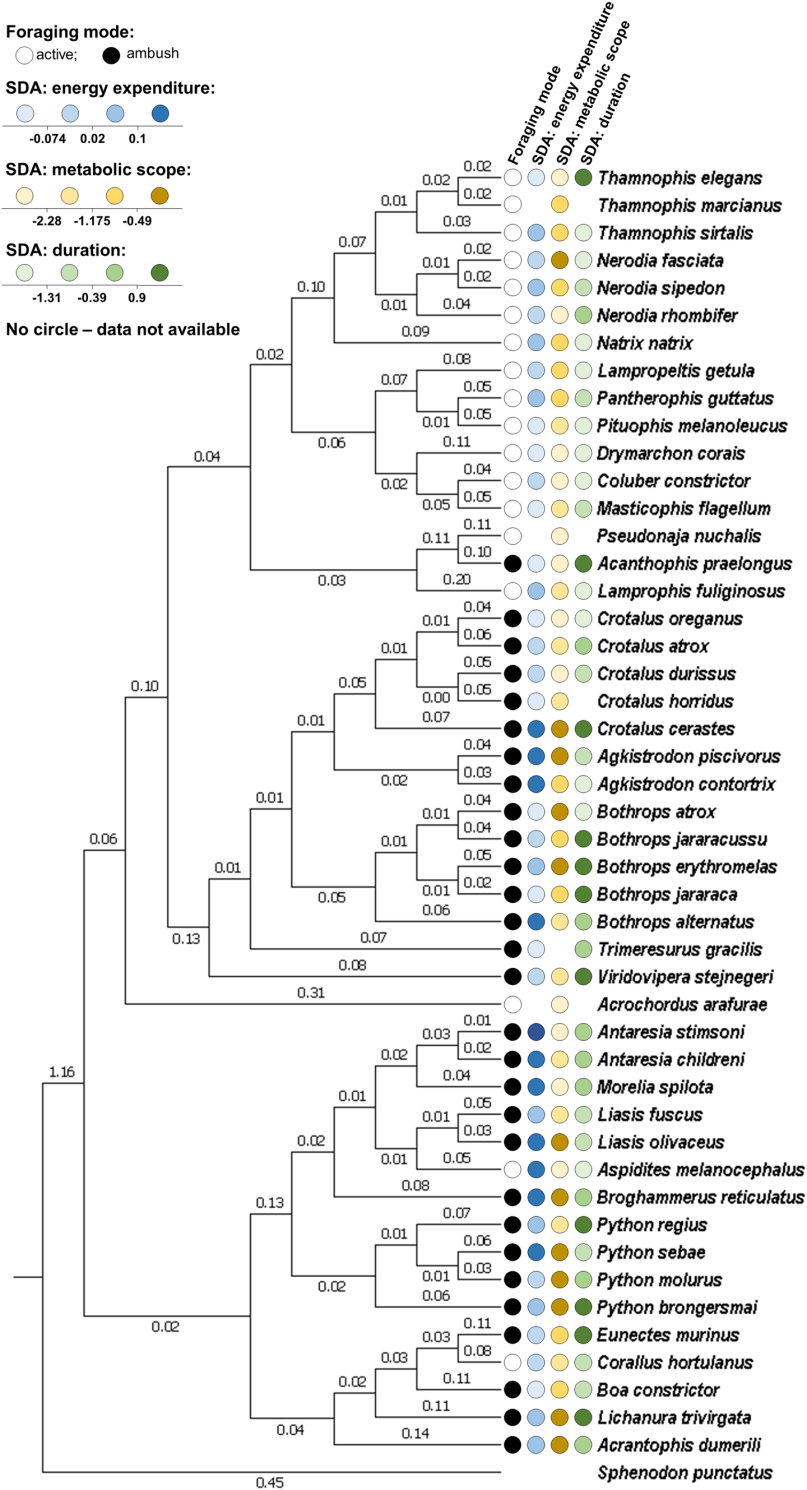
Phylogenetic tree of snakes used in the study obtained from Pyron et al. (2013). Tuatara (*Sphenodon punctatus*) is used as outgroup. Values of branch lengths are displayed. SDA variables are presented as residual values independent to the effects of a meal size, snake body mass and measurement temperature (see Materials and methods section)

**Fig. 3:**
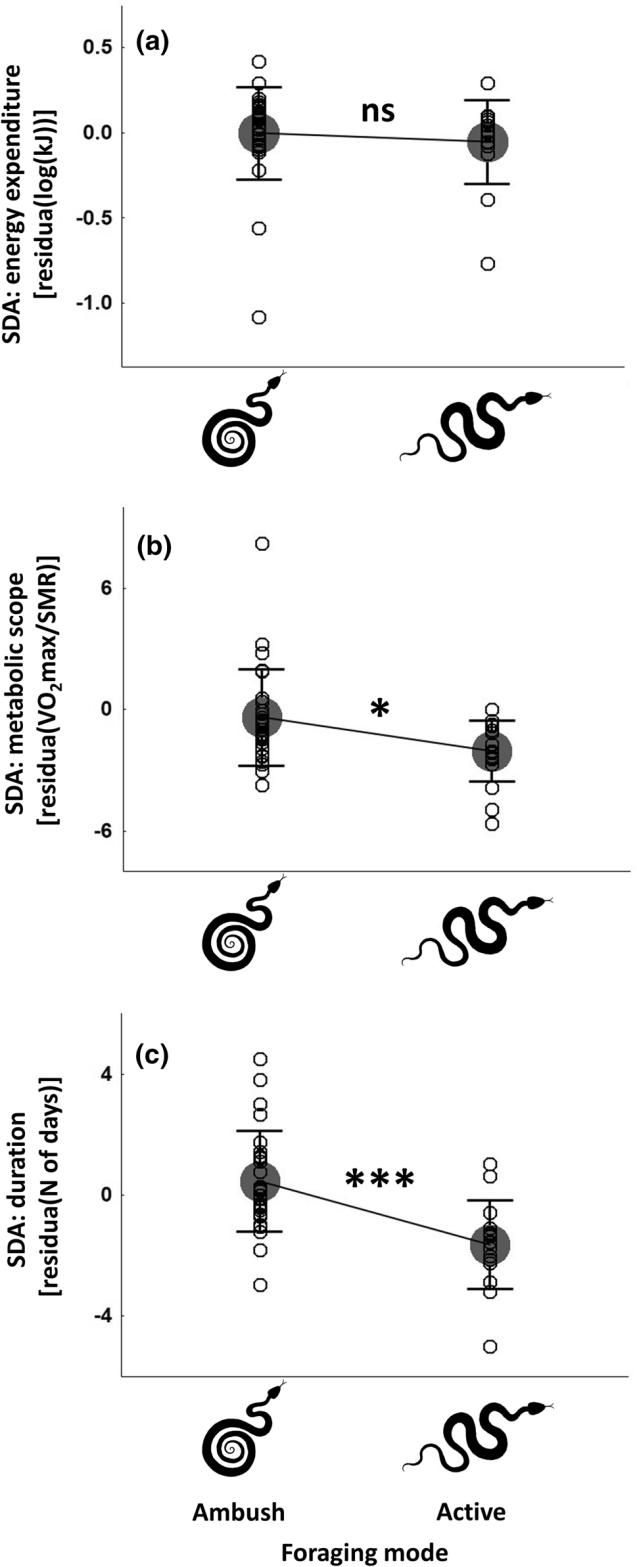
Relationships between foraging mode (ambush vs active) and three variables describing Specific Dynamic Action (SDA) in snakes: **a** SDA energy expenditure, *p* = 0.88; **b** SDA metabolic scope, *p* = 0.035; **c** SDA duration, *p* = 0.00021. Each variables is expressed as a residual value independent to the effects of a meal size, snake body mass and measurement temperature (see Materials and methods section). Grey dots represent mean; open circles represent single species; whiskers indicate standard deviations; asterisks indicate level of significance

My study provides evidence that SDA does vary in relation to foraging mode (Figs. 2, 3) and can thus be considered to correspond with the foraging mode syndrome (Beaupre and Montgomery 2007). The effect of foraging mode was, however, not ubiquitous among the variables describing the costs of SDA. Specifically, the SDA_expenditure_ appeared to be unrelated to the foraging mode (Fig. 3a), whereas the SDA_scope_ (Fig. 3b) and SDA_duration_ (Fig. 3c) both reached higher levels in ambush than in active predators. This effect was evident even after controlling for additional factors known to strongly impact SDA response, i.e. relative meal size, snake body mass and temperature. An alternative pattern, that is lower values of SDA characteristics in ambush predators, would indicate an adaptation to attenuate the costs of regular ingestion of a larger meals (Secor et al. 1994). This, however, has not been observed, not surprisingly given that the size of average prey has recently been shown not to represent a substantial attribute of foraging mode in snakes (see Glaudas et al. 2019). Greater instead of smaller values of SDA variables in ambush predators indicate, therefore, that foraging-mode specificity of SDA have evolved in response to differential feeding frequency associated with different magnitude of gut remodelling (‘pay-before-pumping’ Secor and Diamond 1995) rather than an average meal size. The effect of foraging mode on SDA was, however, non-uniform among all SDA variables, which is an unexpected result, and suggests that the predictions of the energetic costs of digestion being higher in ambush than in active predators derived from the ‘pay-before-pumping’ concept (Secor and Diamond 2000, 1995) have only partially been met in my results and, thus, need to be revisited. I, therefore, propose that foraging modes in snakes have not coevolved with different size of the entire SDA energy budget (SDA_expenditure_); instead, this coevolution has been with the different size of some of its components manifested in the SDA_scope_ and SDA_duration_.

### SDA response and postprandial physiological upregulation

The ‘pay-before-pumping’ hypothesis states that the vast structural upregulation of the gastrointestinal system after ingesting a meal entails a high energetic cost, being higher when feeding frequency expressed by a species is lower (Secor and Diamond 1995; Secor et al. 1994). The lack of a detectable effect of foraging mode on the total SDA_expenditure_ (Fig. 3a) could indicate that the magnitude of visceral upregulation is comparable among foraging modes, despite different feeding frequencies, or else that its costs are negligible. The contribution of gut mobilization to the SDA response is debatable for two main reasons (Wang and Rindom 2021). First, Overgaard et al. (2002) suggested that if the costs of intestinal growth were a significant part of SDA, then a meal provided shortly after the previous one should result in a reduced SDA response because the intestines are already activated. On this basis, it was suggested that the costs of intestinal hypertrophy were minor, given that no decrease in SDA_expenditure_ was detected in pythons (*Python molurus*) following the ingestion of a meal after only 3 days of fasting (Overgaard et al. 2002), a period of time when intestines are assumed to remain enlarged (Starck and Beese 2001). However, these latter authors used snakes of a greater body mass, which were kept at a lower temperature and fed with comparable or larger meals compared to Overgaard et al. (2002). These factors are known to affect SDA dynamics (e.g. Zaidan and Beaupre 2003, this study), so the downregulation of the intestines could have been triggered earlier in the snakes used by Overgaard et al. (2002). In contrast to the study by Overgaard et al. (2002), other research on a sit-and-wait snake, the timber rattlesnake (*Crotalus horridus*), has shown that a shorter fasting period reduces the costs of subsequent digestion (Zaidan and Beaupre 2003). It, therefore, seems premature to draw conclusions about the inexpensiveness of gut upregulation based on the fasting effect.

The second argument that questions the level of costs incurred by physiological upregulation is that the growth of intestinal mass is proposed to be driven mainly by cell swelling rather than cell proliferation (Starck and Beese 2001). Any increase in intestinal mass must, however, be accompanied by active transportation and/or synthesis of the intracellular content, both known to incur energetic costs (Livesey 1984; Mount 1978; Essig and Caplan 1968). In turn, enlargement of the microvilli indicates enhanced amounts of cellular membranes, the synthesis and maintenance of which are costly (Kozlowski et al. 2003). Importantly, not only is the intestinal mass upregulated, so are a wide range of structures and proteins, including enzymes, transporters, organs other than the small intestine etc. (Wang and Rindom 2021; Secor and Diamond 2000; Starck and Beese 2001). In conclusion, even though changes in particular structures do not appear to incur high metabolic costs, the cumulative expenditure of complete physiological remodelling following ingestion may not be negligible, even if it represents only a minor part of SDA. Although this upregulation is probably underpinned by a similar mechanism in different snake taxa (Starck and Beese 2002), the changes in visceral morphology and physiology has been convincingly recorded as being greater in ambush than in active predators (Secor and Diamond 2000; Cox and Secor 2010). Consequently, the range of increase in metabolic rate should also be higher in ambush predators, a pattern that this study has revealed (SDA_scope_, Fig. 3b). The wide range of structural mobilization driven by infrequent feeding in ambush predators also indicates that more time is needed before the viscera achieve a maximum level of functional performance following the ingestion of prey and, potentially, to return to quiescence on completion of digestion. The significance of foraging mode in the temporal dimension of SDA has already been raised, i.e. the initiation of meal processing has indeed been reported slower in ambush than in active predators (Secor and Diamond 2000; Secor 2003). These findings provide a plausible mechanism for explaining the reported here longer SDA_duration_ in infrequently feeding ambush than in frequently feeding active predators (Fig. 3c).

Why does the total SDA_expenditure_ not depend on the foraging mode, despite the conspicuous foraging-mode driven variation in SDA_scope_ and SDA_duration_? As stated earlier, even if the costs of physiological upregulation vary in relation to foraging mode, they probably do not represent a large part of the entire SDA response (Wang and Rindom 2021), perhaps not large enough to elicit a detectable variation in the entire energy budget expended on digestion (SDA_expenditure_) between ambush and active hunters. Second, even though structural upregulation may consume more time and energy in ambush predators, it leads to superior functional performance, as indicated, e.g. by the higher rate of intestinal nutrient uptake (Cox and Secor 2010; Secor and Ott 2007; Secor 2001). Conceivably, the costs incurred for structural remodelling could be balanced, because their functions are performed more efficiently and/or require a lower turnover rate (Koehn 1991), thus potentially less energy is required. Such an optimization could represent a hypothetical adaptive mean to avoid excessive costs of digestion by infrequently foraging ambush predators.

### Broader implications

With few exceptions, snakes do not comminute their prey (e.g. Jayne et al. 2002). Instead, by swallowing their prey whole, snakes consume exceptionally large meals, commonly reaching 20% and even more than 100% of their body mass (Glaudas et al. 2019). Consequently, meal size appears to be an important predictor of the total costs of digestion (e.g. Toledo et al. 2003; Zaidan and Beaupre 2003). This study has confirmed that each of the tested SDA variables correlates significantly with meal size. Interestingly, no such relationship was found for the effect of temperature, which was significant only for the SDA_duration_, but not for the SDA_scope_ and SDA_expenditure_, despite the known effect of temperature on metabolic rates in snakes (Lillywhite 1987). This implies that the same amount of energy is being expended for a given meal size, regardless of temperature, except that digestion may take longer in colder conditions. Like previous studies, the present one also indicates that particular components of the energy budget display different levels of thermal sensitivity: SMR and activity metabolism are more strongly dependent on temperature (Bury et al. 2018; Hailety and Davies 2009; Bennett and Gleeson 1976) than SDA (this study). Because of the differently composed annual energy budget, one can, therefore, predict that ambush and active predators respond differently towards ambient temperature change, e.g. due to climate warming. The relative contribution of temperature-sensitive SMR to the energy budget is higher in ambush predators, probably because of lower energy expenses on movements, but the absolute amount of energy consumed for SMR is greater in active predators (Secor and Nagy 1994). Through the effect of temperature on SMR, rising ambient temperatures could drive a faster increase in the total energy expenditure of active predators and elevate their already high food requirements (Bury et al. 2018; Beaupre and Montgomery 2007). Increased food requirements are, however, unlikely to be met by the current availability of resources in the environment, because these are predicted to shrink (Huey and Kingsolver 2019). Collectively, therefore, the susceptibility to the ‘metabolic meltdown’ effect (Huey and Kingsolver 2019) may be greater in active than in ambush predators.

## Supplementary Information

Below is the link to the electronic supplementary material.

Supplementary file1 (XLSX 46 KB)

## Data Availability

Data are available in a Supplementary File.

## References

[CR1] Abrams PA (1982). Functional responses of optimal foragers. Am Nat.

[CR2] Abrams PA (1991). Life history and the relationship between food availability and foraging effort. Ecology.

[CR3] Beaupre SJ, Montgomery CE, Reilly SM, McBrayer LD, Miles DB (2007). The meaning and consequences of foraging mode in snakes. Lizard ecology: the evolutionary consequences of foraging mode.

[CR4] Bedford GS (1996) Metabolic physiology, digestive efficiency and energetics of some Australian pythons. MSc dissertation, Northern Territory University, Darwin

[CR5] Bennett AF, Gleeson TT (1976). Activity metabolism in the lizard *Sceloporus occidentalis*. Physiol Zool.

[CR6] Bessler SM, Stubblefield MC, Ultsch GR, Secor SM (2010). Determinants and modeling of specific dynamic action for the common garter snake (*Thamnophis**sirtalis*). Can J Zool.

[CR7] Bury S (2021). Sex-specific growth is mirrored in feeding rate but not moulting frequency in a sexually dimorphic snake. Sci Nat.

[CR8] Bury S, Cichoń M, Bauchinger U, Sadowska ET (2018). High oxidative stress despite low energy metabolism and vice versa: insights through temperature acclimation in an ectotherm. J Therm Biol.

[CR9] Chu CW, Tsai TS, Tsai IH, Lin YS, Tu MC (2009). Prey envenomation does not improve digestive performance in Taiwanese pit vipers (*Trimeresurus**gracilis* and *T. **stejnegeri**stejnegeri*). Comp Biochem Physiol A.

[CR10] Cox CL, Secor SM (2010). Integrated postprandial responses of the diamondback water snake, *Nerodia**rhombifer*. Physiol Biochem Zool.

[CR11] Darwin C (1859). On the origin of species by means of natural selection.

[CR12] Dulloo AG, Girardier L (1990). Adaptive changes in energy expenditure during refeeding following low-calorie intake: evidence for a specific metabolic component favoring fat storage. Am J Clin Nutr.

[CR13] Dunham AE (1978). Food availability as a proximate factor influencing individual growth rates in the iguanid lizard *Sceloporus merriami*. Ecology.

[CR14] Dupoué A, Brischoux F, Lourdais O (2017). Climate and foraging mode explain interspecific variation in snake metabolic rates. Proc R Soc B.

[CR15] Essig A, Caplan SR (1968). Energetics of active transport processes. Biophys J.

[CR16] Even PC, Nicolaïdis S (1993). Adaptive changes in energy expenditure during mild and severe feed restriction in the rat. Br J Nutr.

[CR17] Freckleton RP, Harvey PH, Pagel M (2002). Phylogenetic analysis and comparative data: a test and review of evidence. Am Nat.

[CR18] Glaudas X, Glennon KL, Martins M, Luiselli L, Fearn S, Trembath DF, Jelić D, Alexander GJ (2019). Foraging mode, relative prey size and diet breadth: a phylogenetically explicit analysis of snake feeding ecology. J Anim Ecol.

[CR19] Grafen A (1989). The phylogenetic regression. Philos Trans Roy Soc B.

[CR20] Greene S, McConnachie S, Secor S, Perrin M (2013). The effects of body temperature and mass on the postprandial metabolic responses of the African egg-eating snakes *Dasypeltis **scabra* and *Dasypeltis **inornata*. Comp Biochem Physiol A.

[CR21] Großmann J, Starck M (2006). Postprandial responses in the African rhombic egg eater (*Dasypeltis **scabra*). Zoology.

[CR22] Hailety A, Davies PMC (2009). Effects of size, sex, temperature and condition on activity metabolism and defence behaviour of the viperine snake, *Natrix**maura*. J Zool.

[CR23] Harshman LG, Hoffmann AA, Clark AG (1999). Selection for starvation resistance in *Drosophila melanogaster*: physiological correlates, enzyme activities and multiple stress responses. J Evol Biol.

[CR24] Ho LST, Ane C, Lachlan R, Tarpinian K, Feldman R, Yu Q, van der Bijl W (2018). Package ‘phylolm’. http://cran.r-project.org/web/packages/phylolm/index.html

[CR25] Huey RB, Kingsolver JG (2019). Climate warming, resource availability, and the metabolic meltdown of ectotherms. Am Nat.

[CR26] Jayne B, Voris H, Ng P (2002). Snake circumvents constraints on prey size. Nature.

[CR27] Koehn RK (1991). The cost of enzyme synthesis in the genetics of energy balance and physiological performance. Biol J Linn Soc.

[CR28] Kozlowski J, Konarzewski M, Gawelczyk AT (2003). Cell size as a link between noncoding DNA and metabolic rate scaling. PNAS.

[CR29] Lillywhite HB (1987) Temperature, energetics, and physiological ecology. In: Seigel RA, Collins JT, Novak SS (eds) Snakes: ecology and evolutionary biology. The Blackburn Press, pp 422–477

[CR30] Lindström Å, Enemar A, Andersson G, von Proschwitz T, Nyholm NEI (2005). Density-dependent reproductive output in relation to a drastically varying food supply: getting the density measure right. Oikos.

[CR31] Livesey G (1984). The energy equivalents of ATP and the energy values of food proteins and fats. Br J Nutr.

[CR32] Lourdais O, Gartner GEA, Brischoux F (2014). Ambush or active life: foraging mode influences haematocrit levels in snakes. Biol J Linn Soc.

[CR33] Møller AP, Christe Ph, Erritzøe J, Mavarez J (1998). Condition, disease and immune defence. Oikos.

[CR34] Mount LE (1978). Heat transfer between animal and environment. Proc Nutr Soc.

[CR35] Nespolo RF, Franco M, Franco M (2007). Whole-animal metabolic rate is a repeatable trait: a meta-analysis. J Exp Biol.

[CR36] Overgaard J, Andersen JB, Wang T (2002). The effects of fasting duration on the metabolic response to feeding in *Python **molurus*: an evaluation of the energetic costs associated with gastrointestinal growth and upregulation. Physiol Biochem Zool.

[CR37] Paradis E et al (2021) Package ‘ape’. https://cran.r-project.org/web/packages/ape/index.html

[CR38] Pyron RA, Burbrink FT, Wiens JJ (2013). A phylogeny and revised classification of Squamata, including 4161 species of lizards and snakes. BMC Evol Biol.

[CR39] Reading CJ, Luiselli LM, Akani GC, Bonnet X, Amori G, Ballouard JM, Filippi E, Naulleau G, Pearson D, Rugiero L (2010). Are snake populations in widespread decline?. Biol Lett.

[CR40] Secor SM (2001). Regulation of digestive performance: a proposed adaptive response. Comp Biochem Physiol A.

[CR41] Secor SM (2003). Gastric function and its contribution to the postprandial metabolic response of the Burmese python *Python**molurus*. J Exp Biol.

[CR42] Secor SM (2009). Specific dynamic action: a review of the postprandial metabolic response. J Comp Physiol B.

[CR43] Secor SM, Diamond J (1995). Adaptive responses to feeding in Burmese pythons: pay before pumping. J Exp Biol.

[CR44] Secor SM, Diamond JM (2000). Evolution of regulatory responses to feeding in snakes. Physiol Biochem Zool.

[CR45] Secor SM, Nagy KA (1994). Bioenergetic correlates of foraging mode for the snakes *Crotalus**cerastes* and *Masticophis** flagellum*. Ecology.

[CR46] Secor SM, Ott BD (2007) Adaptive correlation between feeding habits and digestive physiology for boas and pythons. In: Henderson RW, Powell R (eds) Biology of the boas and pythons. Eagle Mountain, Eagle Mountain, UT, pp 257–268

[CR47] Secor SM, Stein ED, Diamond J (1994). Rapid upregulation of snake intestine in response to feeding: a new model of intestinal adaptation. Am J Physiol Gastr L.

[CR48] Speakman JR, McQueenie J (1996). Limits to sustained metabolic rate: the link between food intake, basal metabolic rate, and morphology in reproducing mice, *Mus**musculus*. Physiol Zool.

[CR49] Starck JM, Beese K (2001). Structural flexibility of the intestine of Burmese python in response to feeding. J Exp Biol.

[CR50] Starck JM, Beese K (2002). Structural flexibility of the small intestine and liver of garter snakes in response to feeding and fasting. J Exp Biol.

[CR51] Stuginski DR, Navas CA, de Barros FC, Camacho A, Bicudo JEPW, Grego KF, de Carvalho JE (2018). Phylogenetic analysis of standard metabolic rate of snakes: a new proposal for the understanding of interspecific variation in feeding behavior. J Comp Physiol B.

[CR52] Stuginski DR, Navas CA, de Barros FC, Grego KF, Martins M, de Carvalho JE (2018). The role of feeding specialization on post-prandial metabolic rate in snakes of the genus *Bothrops*. Zool Sci.

[CR53] Szarski H (1983). Cell size and the concept of wasteful and frugal evolutionary strategies. J Theor Biol.

[CR54] Toledo LF, Abe AS, Andrade DV (2003). Temperature and meal size effects on the postprandial metabolism and energetics in a boid snake. Physiol Biochem Zool.

[CR55] Tsai T-S, Lee H-J, Tu M-C (2008). Specific dynamic action, apparent assimilation efficiency, and digestive rate in an arboreal pitviper, *Trimeresurus**stejnegeri*. Can J Zool.

[CR56] Wang T, Rindom E (2021). The physiological response to digestion in snakes: a feast for the integrative physiologist. Comp Biochem Physiol A.

[CR57] Warner DA, Bonnet X, Hobson KA, Shine R (2008). Lizards combine stored energy and recently acquired nutrients flexibly to fuel reproduction. J Anim Ecol.

[CR58] Webb JK, Brook BW, Shine R (2003). Does foraging mode influence life history traits? A comparative study of growth, maturation and survival of two species of sympatric snakes from south-eastern Australia. Austral Ecol.

[CR59] Werner EE, Anholt BR (1993). Ecological consequences of the trade-off between growth and mortality rates mediated by foraging activity. Am Nat.

[CR60] Zaidan F, Beaupre SJ (2003). Effects of body mass, meal size, fast length, and temperature on specific dynamic action in the timber rattlesnake (*Crotalus**horridus*). Physiol Biochem Zool.

